# Impact of Precision Medicine on Drug Repositioning and Pricing: A Too Small to Thrive Crisis

**DOI:** 10.3390/jpm8040036

**Published:** 2018-11-05

**Authors:** Venkataswarup Tiriveedhi

**Affiliations:** 1 Department of Biological Sciences, Tennessee State University, 3500 John A Merritt Blvd, Nashville, TN 37209, USA; vtirivee@tnstate.edu; Tel.: +1-615-963-5779; Fax: +1-615-963-5747; 2 Department of Pharmacology, Vanderbilt University, Nashville, TN 37235, USA

**Keywords:** precision medicine, value-based pricing, drug repositioning, differential value, cost efficiency, healthcare

## Abstract

The pricing of targeted medicines continues to be a major area of contention in healthcare economics. This issue is further complicated by redefining the role of molecular testing in precision medicine. Currently, whilst pricing of clinical laboratory diagnostics is cost-based, drug pricing is value-based. The pricing for molecular testing is under pressure to change the traditional business model, for it has a critical subsidiary role in determining the final value of targeted medicines. The market size for drugs is reduced by molecular testing when patients with the same disease are stratified based on their genetics, it is critical to determine the value of this new enhanced drug specificity to realize its full pricing potential. However, these value-based pricing strategies require a careful understanding of changing market conditions, especially, in the context of stratified patient segments made possible by precision medicine. In this article, we discuss the various factors impacting pricing decisions, and consider evolving economic trends in precision medicine.

## 1. Personalized Medicine

The concept of targeting health care interventions to patients as subsets within the same disease cohort based on a unique set of genetic and biomarker characteristics is referred to as personalized medicine. Multiple names such as precision, individualized, targeted, and stratified medicine are mentioned in the literature, albeit with some minor differences in the exact definition of these terms [1]. However, it is the application of genetics-based diagnostic testing as a companion to clinical laboratory testing, to identify and stratify a select subgroup of patients for targeted delivery of a pharmaceutical medicine or therapeutic procedure.

Drug repurposing refers to finding novel therapeutic indications for already approved, discontinued, and archived drugs, as well as drugs currently undergoing clinical trials [2]. While repurposing is an important issue and requires much basic biomedical and clinical translational research, in the current article, we confine our scope to drug repositioning [3]. We will define drug repositioning as the change in drug of choice (DOC), as determined by patient genetics. As a consequence of this targeted therapy, the traditional first-line drug for a disease could change, and the second or third line drugs could now become the first-line therapy based on the specific genetics of a patient. For example, a drug which is generally considered third-line therapy for hypertension, can now, based on genomic testing evidence, become the first-line drug of choice for that hypertensive patient. This requires serious consideration of the implications of personalized medicine on drug repositioning. While the classical drug pricing model follows a shotgun approach with a wider customer base [4], the emergence of highly-targeted drug therapies will require new pricing strategies to address the case for drug repositioning, such that a highly-targeted drug in a small eligible patient population will carry a much higher probability of being effective. Given the power of regulatory agencies and the role of reimbursement mechanisms to control drug pricing, it is important to redefine and quantify the value of these seemingly new emerging and redesigned therapies, especially, in the context of the probable absence of reference price points. Personalized medicine will have a rocky start with reduced markets, unless a clear understanding of value addition to customers and profit maximization to pharmaceutical companies, along with the potential complementary role of clinical laboratories as providers of molecular diagnostics, is quickly established. In this review article, we will discuss the framework of value justification and changing pricing strategies, highlighting complex trade-offs that must be managed between innovation and diagnostic testing versus value-based pricing.

## 2. Value-Based Pricing in Precision Medicine

While the original intentions of precision medicine were its assumed beneficial financial impact through the development and use of therapies in biomarker-defined patient subcohort populations, real world experiences seem to suggest the opposite. Prices for such targeted therapies have been quoted as high as US $350,000 per patient per year [5,6]. In spite of their high costs, some of these targeted therapies have demonstrated, at least in small limited clinical trials, dramatically improved survival, whilst others have shown minimal benefit [7]. An assessment of value-based pricing is crucial to ensure the payer and reimbursement decision-makers feel comfortable to use precision therapies. In addition, it is imperative for policy-makers and public funding agencies to determine the opportunity cost of research in this direction, resulting from a forgone benefit of investment in other healthcare avenues. 

Peter Drucker noted that cost-based pricing is one of the deadly business sins [8]. In the past two decades, value-based pricing is considered the standard business norm in all fields, including the healthcare industry. Despite the prevailing perception of the pharmaceutical industry as exercising a predatory pricing approach, the fundamental rules of drug pricing follow a standard set of economic rules like any other industry. The limits on a viable pricing range are determined by the market and pharmaceutical industries. Specifically, in the context of precision medicine, drug pricing is further determined by clinical laboratories through biomarker testing, which changes the value of the drug. The market or demand side of the pricing analysis primarily assesses the value of the product as perceived by the customers, in the context of a competitive, free-trade environment. This usually tends to establish the upper-limit of the price range for a product. A company perspective assesses the cost of production, including research and development (R&D), drug development, preliminary phase trials, return on investment (ROI), and net present value (NPV) net analysis to determine a viable price range for the product. This usually sets the lower-limit of the price range for a product. 

An important effect of the value-based approach is that the pricing strategy should capture the potential value that will be generated by the drug, and therefore, this should be reflected in the product development decisions [9]. A generic analysis to capture the perceived value (V) will be the sum (V = A + D) of the cost of the best alternative or reference price (A) plus the differential value of the targeted therapy (D). While the reference or best alternative price is indexed and reasonably established, the key final price determinant is the value addition perceived by patients and their representative payers towards the product, as established by molecular testing of patient genetics. In personalized medicine, there will be a significant shrinkage of the customer base for a product, but simultaneously, there will be an enormous increase in the specificity and success of the product, which will translate into heightened differential value perception by payers. 

The question of defining the product in personalized medicine during drug development is not simple. Drug development in personalized medicine starts with a molecule that might have a strong impact on highly stratified patient groups, with specific indications that are dependent on the line of therapy, target patient segments, treatment setting, or mode of administration. These variables determine the differential value, drug repositioning, and pricing opportunity. Furthermore, a previous conventional pharmaceutical industry strategy might require a 180 degree shift of thinking. An example of this effect on drug positioning and pricing is an appeal by Novartis regarding usage of pimecrolimus for atopic dermatitis. It involves a previous recommendation by the National Institute for Clinical Excellence (NICE) (United Kingdom) to endorse tacrolimus (Prograf, Fujisawa Pharmaceutical, Tokyo, and Japan), but not pimecrolimus (Elidel, Novartis, Basel, and Switzerland), for the treatment of atopic eczema. Although pimecrolimus was licensed for first-line use in patients with mild-to-moderate disease, its benefit over the reference product of topical corticosteroids was not considered to provide good value for money. However, tacrolimus was approved as a second-line treatment for moderate-to-severe disease, which translates into a positioning advantage, where the differential value over the use of the reference drug, i.e., high-dose corticosteroids were considered to be worth a higher price, which pharmaceutical industries wanted to capture. However, following an appeal by Novartis, pimecrolimus was approved for second-line treatment, which represents industrial success towards promoting a higher profitability product [10]. This highlights the importance of conceiving well-defined product scenarios from an industry angle, considering the pricing and value implications of potential drug positioning in the treatment regimen and different stratified patient populations’ early in the drug development.

## 3. Shift from Experience Goods to Search Goods

An important reason for drug repositioning by personalized medicine is the conversion of pharmaceutics from ‘experience goods’ to ‘search goods’. In the current empirical medicine-based healthcare structure, healthcare providers (physicians and hospitals) work with their patients to try an initial set of therapies (first line drugs) based on their proven efficacy from population-based studies. Based on the experience from these first line drugs, the realized efficacy, and side-effects on patients, the next step in therapy is recommended. This strategy, although scientific, has a trial and error flavor making the drugs seem like ‘experience goods’, where their efficiency can be determined only after consumption. However, personalized medicine, by adding companion genetics based diagnostic testing, will recommend a drug which suits a given patient, and thus converting pharmaceutics to ‘search goods’, whose efficiency can be predicted before consumption [11]. This paradigm shift in pharmaceutics from experience goods to search goods could drastically reduce healthcare costs, such as the length of hospital stay, and thereby reduce the financial burden of a disease, specifically, chronic diseases and cancers, which incur high costs from prolonged inpatient stay. 

This paradigm-shift also plays an important behavioral change in patient’s adherence to healthcare. As noted by Egan and Philipson (2015) [12] and Sood et al. (2013) [13], a strong correlation exists between patient’s adherence to healthcare and the perceived value of personalized medicine. Adherence is defined as the patient’s belief in healthcare as determined by prior experience with the line-of-therapy previously provided to the patient. This line-of-therapy, if based on population-based evidence, has higher chances of failure and results in patient nonadherence to healthcare. However, if this line-of-therapy is tailored to a patient with companion diagnostics, it will have higher chance of patient adherence to healthcare [14]. While assessing the value of personalized medicine, it is critical to quantify this factor of patient adherence, which could be another interdependent factor regarding patients attitudes towards risk aversion.

While the market for targeted medicine is rapidly expanding, it is important to quantitatively determine the interdependency between molecular testing and drug development in precision medicine. To realize the full value of precision medicine for both molecular testing and therapeutics, we should carefully perform a cost-benefit analysis. There would be obvious challenges in performing these analyses, especially, given the complexity and intrinsic uncertainty in assessing tests with multiple biomarkers, along with heterogeneity across cancer types and stages in various subcohorts of the same disease. Retrospective evidence to allude this complexity involves a clinical trial with gefitinib, for the treatment of ovarian cancer, where only 1 out of 27 patients responded, which by most clinical trial standards would considered as a drug failure. However, this was the only patient with a mutation in epidermal growth factor receptor (EGFR) molecule, which was the target of this drug [15]. Similarly, in oncology, pharmacogenetics testing of dihydropyrimidine dehydrogenase deficiency could be used to assess the successful application of 5-fluorouracil-based chemotherapy [16]. This example suggests a careful look at previously assumed clinical trial failed drugs in the light of precision medicine. This is further compounded by relatively minimal evidence on the actual survival benefit of these targeted therapies. Despite these challenges, economic evaluation is needed to determine the potential value by manufacturers, governments, patients, and payers for reimbursement decisions. 

Another challenge in determining the value of the drug in precision medicine is that different indications generally involve distinct customers, value propositions, competition (also called reference) for products, and different doses for the same drug [17]. Although the value-based approach might theoretically justify appreciably different prices for each indication, in reality, it is not viable to achieve large price spreads in a given geographic territory for the same drug on the basis of differing indications, unless a differing dose relationship supports this claim. Some strategies that could be adopted include developing a higher-priced launching indication ahead of a lower-priced one, thereby, securing a higher-priced ‘anchor point’ for subsequent indications; targeting the product in lower-priced segment for a patient subpopulation with severe disease and thereby securing higher value; and developing different formulations (for example, tablets versus injections) for different indications to enhance the possibility of different prices of each separate therapeutic indication. Furthermore, most countries do not allow a price rise once they are initially set by a company. The challenge for pharmaceutical companies is finding a trade-off between launching a new targeted therapy at a lower price with limited R&D versus launching later at a higher price following the results of extensive long-term clinical studies.

A reasonably well agreed upon area of value-addition by these targeted therapies is their unique ability to reduce inpatient hospital stay. For example, in 2015, the United Kingdom spent approximately £1.3 billion (US $1.83 billion) on inpatient hospital services for patients in their last year of life. Similarly, Australia spent approximately 79% (AU $3.6 billion, US $2.6 billion) and the USA spent 38% (US $31.3 billion) of its total cancer expenditure on inpatient hospital services [18,19]. While still speculative, there is convincing evidence that pharmacogenetics testing significantly reduces the hospitalization and emergency department visits which would argue in favor of value addition to precision medicine. 

## 4. Choosing Correct Reference for New Molecular Entities

In the context of value-based pricing for drugs in personalized medicine, it is critical to establish an appropriate reference to justify the higher prices. The reference therapeutic option, and therefore the reference price for a new product, depends primarily on its intended indication and its placement in the treatment regimen. The reference therapeutic option is generally the current standard of care. If there is no contemporary drug therapy for a condition then the new drug could be compared to an expensive surgical or radiotherapy procedure in cancer. Furthermore, if there has been no pharmaceutical innovation in the disease area, such as malaria, and the current standard of care is old, generic, or cheap, it is the responsibility of the manufacturers to explain that the high cost of the new drug represents substantial positive differential value to secure pricing commensurate with an innovative product. Similarly, if the side-effects and resulting repeated hospitalization profile for the new product are significantly lower than the older generic medication, reduced healthcare costs can be successfully demonstrated. This is the case with new atypical antipsychotics that are used against schizophrenia, and therefore, would explain the significantly higher pricing over typical antipsychotics. 

As the reference therapeutic option forms the basis for the value perception of a new product by the buyer, a crucial part of pricing-strategy development is framing the value of the new product in the comparative context of the desired price reference. Undesired comparisons by regulatory agencies could be a major challenge. For example in Europe, products are grouped into common therapeutic categories using the ‘Anatomical Therapeutic Chemical Classification System’ (ATCCS). This would imply that the allowed price reimbursement for the new targeted drug, such as a new immunotherapeutic drug for cancer, would be limited to the average, or the lowest price in that given category. Any difference between the allowed reimbursement amount and the actual drug price must come out of the patient’s pocket. This could be daunting, as most of the immunotherapeutic drugs cost 200 times higher than the traditional chemotherapeutic drugs in the same category. Manufacturers of new innovative personalized medicine products would clearly seek to demonstrate a value difference from existing drugs to avoid being grouped with older cheaper drugs. 

## 5. Assessing Full Differential Value

A reasonable starting point for analyzing the value of new therapy is by performing a well-defined randomized clinical trial. However, the choice of clinical endpoints, inclusion criteria for patients, and the choice of control drugs for statistical comparison play a critical role in the final evaluation of the perceived value of the new product to the relevant potential customers. A well-accepted parameter to quantitate the differential value of a new personalized therapy is by demonstrating a change in health outcome as measured by the change in quality-adjusted life years (QALYs), which captures the well-being as compared to alternative health states with and without adoption of the new therapy. Another parameter used for value analysis is the incremental cost-effectiveness ratio (ICER), as determined by the ratio of the difference between the cost of new and current standard therapy to the difference QALY between new and current standard therapy.

ICER = (Cost of new therapy − Cost of current standard therapy)/(QALYS generated by new therapy − QALYs generated by current standard therapy)

Many countries use this approach to make reimbursement decisions. In the United Kingdom, a threshold of £20,000 per QALY to £50,000/QALY for more life-threatening diseases would be set as the upper limit on the willingness to pay for therapeutic advances. In the US, it may vary from $50,000/QALY to $150,000/QALY depending on the severity of the disease. In contrast, most immunotherapies and other targeted therapies amount to $250,000/year which is much greater than the ceiling amount. Further, it is extremely difficult to capture the full value of targeted therapies for chronic diseases as the full depth of the therapy and potential complication spectrum from extending the lifespan and side-effect profile from prolonged usage of therapy cannot be recognized. This challenge is to circumvent this by using a risk-sharing strategy where partial evidence that a new product has significant value could be used to set a premium launch price pending amendment following a detailed study to determine the long-term outcomes. At the same time, the traditional value analysis by the patient which is dependent upon the parameters such as beneficial to quality-adjusted life-years (QALYs) outcome and use of subjective instruments such as EQ-5D (impact on mobility or pain, etc.) might be irrelevant in precision medicine [20]. In the context of precision medicine, a patient will receive a targeted treatment based on molecular testing. This enhances the certainty of treatment benefit which translates into increase perceived value of precision medicine. 

**Figure 1:**
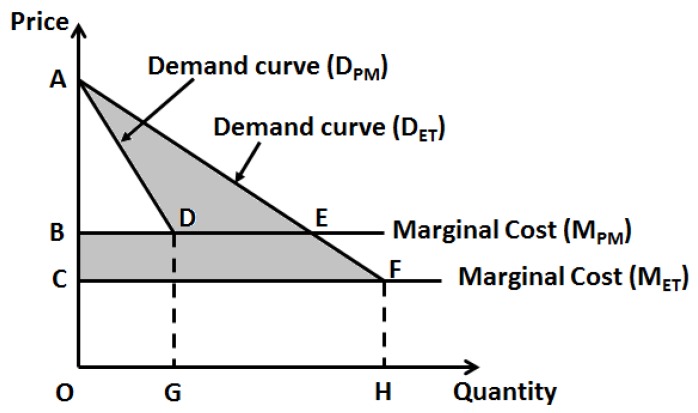
Impact of personalized medicine on total revenues of already existing pharmaceutical treatment entities. M_PM_ refers to the marginal cost following adoption of precision therapy and M_ET_ refers to the marginal cost of the current empirical therapy. ΔABD-total revenue of already existing entity from personalized medicine; A sum of ‘ADE’ and ‘BCFE’ would be the total loss (or lost revenues) from personalized medicine.

**Figure 2:**
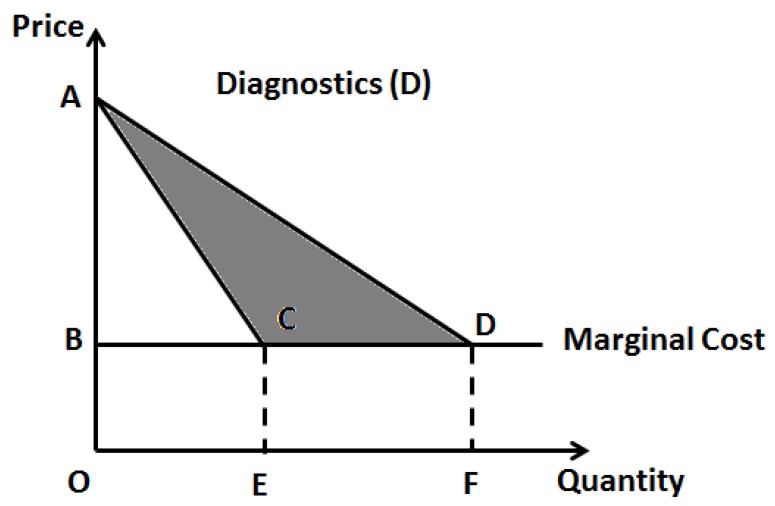
Impact of personalized medicine on total revenues of diagnostic procedures. ΔACD-total additional revenues (or profits) for diagnostic laboratory facilities from personalized medicine.

For already existing pharmaceutical entities, a challenge with drug repositioning is that, unlike in other industries where product pricing is more dynamic and amenable to changes, in healthcare drug pricing is heavily regulated and it is unlikely that a change in drug price would occur after pricing is initially set. However, as personalized medicine reduces the market (quantity demanded) for a drug the marginal costs (and possibly overhead for infrastructure updates) would invariably raise (Figure 1). But given the fact that the price of the pharmaceutical entity does not change, the realized profits (or specifically, total revenue) will decrease. To this effect, Philpson (2018) makes an interesting case for a possible two-part pricing to overcome this challenge [21]. As diagnostic testing is an integral part of the personalized choice of pharmaceutics, it would make sense to combine these two aspects for optimal healthcare pricing. The logic behind this two-part pricing strategy seems obvious as this would enhance profits and innovation incentives when diagnostics is clubbed with the treatment, the lost profits in treatment alone can be recovered from the diagnostics (Figure 2). However, this warrants a merger incentive for joint ownership of the two components, namely, the clinical diagnostic laboratory facility and pharmaceutical drug manufacturing facility, so that the joint owner can exert any kind of monopoly pricing to this combined product as against two separate owners. This strategy will be along the lines a standard economics rule that the monopoly profits of the joint ownership from vertical merger must be higher than the sum of the total profits from the two separate owners [22]. This strategy, although extremely logical, requires more practical evidence as most of the clinical testing facilities are either independently owned or operated under a hospital facility, while, the drug industry is usually a huge separate industry. Most countries have strict rules that healthcare providers (physicians) should not have direct ties (or profit interests) with the pharmaceutics industry, so as not to have bias on therapeutic judgment towards drug selection.

**Figure 3:**
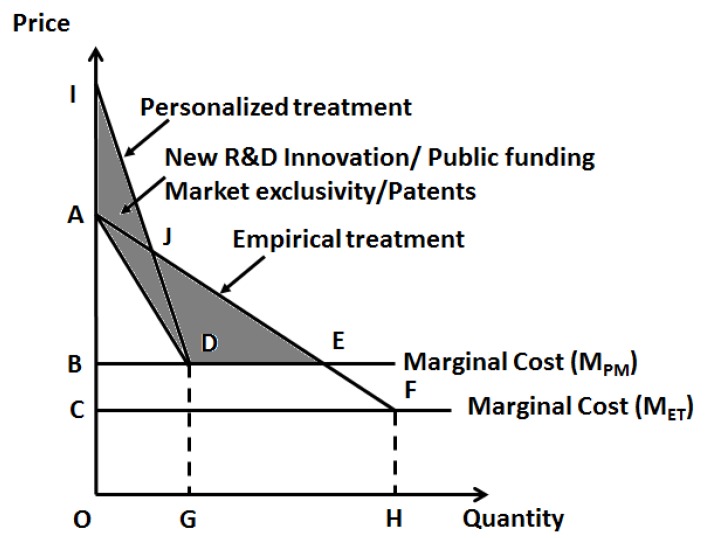
Potential new pricing for new molecular entities compared to empirical pharmaceutical entities. Shaded area is the potential R&D costs (amount) needed to invest upfront to bring down the prices of new molecular entities. This amount should either be recovered from short-term higher pricing of new molecular entities or investment to be done by public funding agencies like the National Institutes of Health in academic institutions to bring down the prices.

On the other hand, for new molecular entities to be added to the market for personalized therapy with higher pricing (Figure 3), it is critical to provide an abundance of evidence in favor of the differential value provided by these newly developed drugs. Here, R&D and innovation plays an important role. As the majority of innovation is led by academic institutions, public agencies, such as the National Institutes of Health (NIH), could provide tax-payer dollars for research to promote R&D and bring down the prices of new pharmaceutical entities.

## 6. The Customer Complexity in Precision Medicine

**Figure 4:**
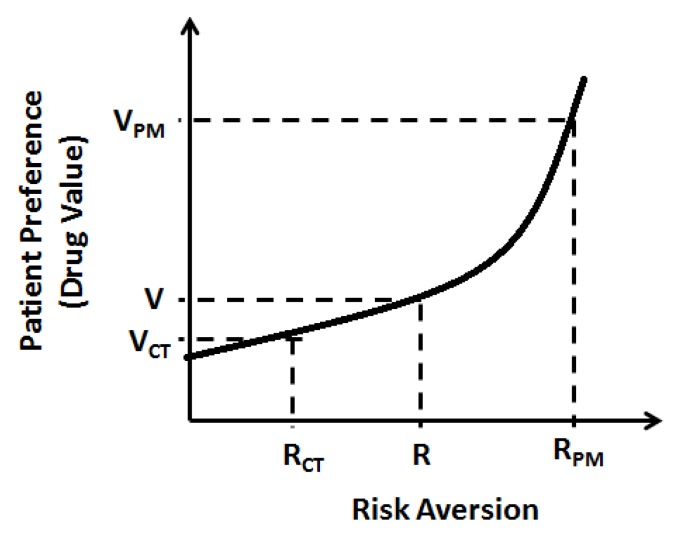
Patient’s perceived differential value to personalized medicine as a consequence of increased risk aversion. V refers to perceived drug value by patient, VPM refers to perceived value of precision medicine, VCT refers to perceived value of current empirical treatment, R refers to patient’s expected risk aversion, RPM refers to risk-aversion by precision medicine, and RCT refers to risk aversion by current empirical treatment.

A key component of value analysis for any therapy is its ability to determine disease risk aversion with or without therapy. For this, the disease risk analysis would be primarily provided by molecular testing which should be considered an integral part of targeted therapy. A personalized treatment could then create additional value for individuals by reducing the uncertainty they have that they will respond towards a given treatment. This risk aversion is critical for calculating the value of precision medicine by the customer (Figure 4). However, unlike in most industries where the consumer initiates purchase of a product, in healthcare, the consumer role is segregated into three parties—the prescriber, the patient, and the payer—that all have an influence over the purchase decision for a particular product. To some degree, each of these three parties has varying value perceptions. However, all countries have several governmental regulations to minimize the bias in product selection pressure between the three parties. 

Priorities for allocation of resources in precision medicine might not be consistent between stakeholders, further complicating value assessment and reimbursement decision-making. The existing evidence strongly suggests that patient preferences are not commonly considered or prioritized by payers/reimbursement agencies or public policy-makers [23]. The most interesting finding is that patients seem to value improvement in quality-of-life and palliative care, rather than prolonged survival; 5-year survival benefit was taken as the bench-mark for therapeutic efficiency of a drug. This preference is further supported by a study from Singapore where patients were willing to pay a premium of around $14,000(USD) for treatment that allowed them to die at home rather than in hospital and $31,000(USD) for treatment that would reduce pain, in comparison to only $8000(USD) for a treatment that extends their life by an additional 12 months [24]. On the contrary, an opposite evidence came from the behavioral studies performed in the US and UK which showed that people thought it was a moral and/or ethical obligation for governments and tax-payers to provide treatments to end-stage metastatic cancer patients [25,26]. This evidence clearly conflicts with the priorities established among various stakeholders acting as customers in healthcare business.

In some countries, such as Germany and the United Kingdom, where the providers (or hospitals) are subject to some degree of healthcare budget responsibility will make the hospitals more price sensitive than in countries such as the United States where the payment responsibility predominantly lies with the insurance agency and patient. An important challenge with having three apparently different self-interest groups as payers is that it will result in an asymmetric distribution of healthcare information. The prevailing ‘silo mentality’ in which drug budgets are generally seen as separate from other healthcare budgets, presents a major challenge to precision medicine wherein the drug usage is dependent upon molecular testing which in turn impacts the inpatient hospital stay and long-term outcomes of a disease. This compartmentalization of healthcare prevents the full appreciation of the value of precision medicine. In general, prescription medicines account for only 10% to 15% of the healthcare costs. The value evaluation of drugs used in precision medicine should, therefore, be seen in the context of the entire healthcare expenditure or rather a potential reduction in the net expenditure.

The reimbursement agencies and government policymakers should decide the opportunity cost of adoption of a new technology by determining the foregone benefit. There have been approximately 600,920 deaths from cancer in the United States in the year 2017 and as a first approximation, if we assume the government decides to allocate US $350,000 for drugs and molecular testing as part of healthcare costs to each of these patients, that would require over US $210 billion (the equivalent 2017 actual cost was 80.2 billion) just to cover the drug costs for this one group of patients for a year [27]. Funding of targeted therapies at such high levels of expenditure will exhaust the entire government welfare schemes budget allocation to oncology precision medicine alone. With the advancement of technology and more targeted therapies entering the market, resource allocation to meet the demand could be a challenge. It is therefore imperative to set priorities on the possible upper-limit of the fundable costs to these targeted therapies. At the same time, there could be a difference in the public preferences and perceptions on precision medicine. Research from a National Institute for Health and Care Excellence (NICE)-led study on public preferences in the UK to determine a potential increase in government funding to end-of-life treatment at a higher threshold limit per QALY gave interesting results. In this survey, approximately 4000 members of the general public in England and Wales indicated that no extra priority should be made for the end-of-life treatment [28]. This is in agreement with the health economists’ contention that such a policy would result in a decrease in public welfare funding to other schemes [29]. 

## 7. The Company Perspective

A careful assessment of the value perceptions of the market early in the product development is critical for generating an adequate ROI over the product life cycle. To recover the sunk costs during the research and development phase, which might extend up to 10 years, a higher gross margin would be needed to establish a positive net present value (NPV) for the product. In an NPV calculation, an appropriate discount rate should be chosen which would represent the opportunity cost of capital. This opportunity cost will be defined as the financial return that could be obtained by investing capital in the next-best alternative investment. A discount rate of 10 to 15% is generally chosen in the pharmaceutical industry as the standard rate at which value to most of the products are developed. The NPV calculation incorporates several kinds of costs including, preclinical basic science research costs, clinical trials, post-launch testing, marketing, and manufacturing scale-up costs. A general rule in pharmaceutical industry is that for every 5000 molecules tested in the laboratory, only five reach Phase I, out of which only one will actually be marketed. The NPV would be calculated and updated continually along the product development timeline as new clinical and market data become available. This is critical for investment and capacity building decisions. The need to make these investment decisions before drug launch, underscores the importance of pricing strategies in precision medicine.

The benefits of molecular testing could be complex and difficult to evaluate with the currently available economic methods, potentially resulting in misrepresentation of their realized value. A broadening of current concepts of value and application of different economic approaches to the assessment of molecular testing would be required to ensure the true value is revealed and to make appropriate resource allocation decisions. Payments in precision medicine would imply payment to diagnostics and payment to therapy. Generally payments to diagnostics are cost-based, while payments to drugs are value-based. However, an interesting complication here is that diagnostics work as complimentary tools towards valuation of targeted therapy drug prices. The traditional logic behind cost-based reimbursement of testing is that testing is not considered to change the health status of a person. However, a new dimension of complexity arises in precision medicine wherein, testing leads to determining the right treatment and changing the path of the patient’s health status. Thus molecular testing in targeted therapy context is acting as a complementary tool to create a market for the new drugs.

## 8. Conclusions 

Advances in modern medicine will result in a desired increase in human life-span along with an undesired increase in costs on expensive technologies. An imbalance will continue to exist between the demands for advanced therapeutic strategies, such as personalized medicine, along with the inability to fund such advanced healthcare measures. Cost containment will be key for the future of personalized medicine. A holistic understanding of healthcare economics is needed in devising the delivery of this new aspect of medicine. Pure cost control measures, such as expenditure through government welfare schemes, might not be a viable option. Overall this is just the beginning of determining the value and affordability of precision medicine. As drug repositioning will be a futuristic reality and an obvious consequence of personalized medicine, it is critical to clearly establish the differential value of the already existing pharmaceutical entities in the market and find a solution for the potentially lost revenues due to shrinking market for these drugs. A potential joint ownership strategy with vertical merger of the diagnostic and pharmaceutical facilities would be a worthwhile option. Hospitals and physicians, as advocates for patients’ interests, should play an active role in negotiating with insurance agencies and public policy agencies to demonstrate the differential value of personalized medicine with respect to reduced inpatient stay and enhanced QALY to recover for the potential losses from reduced markets with adopting personalized medicine. A number of important considerations have been outlined in this review article towards establishing the differential value, including the importance of economic evaluation to determine value and the need to account for opportunity cost in decision-making; the complex benefits of molecular testing and the need for advancements in the economic methods for evaluating molecular diagnostics; and the identification of unique patient and public preferences on funding for precision medicine, all these could lead to resetting or establishing a fair price model for the established and new pharmaceutical entities in the healthcare market. All stakeholders involved in precision medicine should appreciate these issues and contribute to a better understanding of the impact of precision medicine. Only then, the complex code that is the value proposition of precision medicine be revealed. Innovative cost efficiency strategies and novel healthcare economic models involving, hospitals, pharmaceutical industries, and reimbursement agencies are needed for the adoption of precision medicine in patient care.
